# The emerging threat of bioterrorism.

**DOI:** 10.3201/eid0504.990403

**Published:** 1999

**Authors:** J M Hughes

**Affiliations:** Centers for Disease Control and Prevention, Atlanta, Georgia 30333, USA. jmh2@cdc.gov


### The Emerging Threat of Bioterrorism

**James M. Hughes**
Centers for Disease Control and Prevention, Atlanta, Georgia, USA

The threat of bioterrorism focuses attention on overall preparedness to address the challenges posed by new and reemerging infectious diseases. Bioterrorism scenarios illustrate the diversity of disciplines and perspectives required to confront these threats, whether naturally occurring or purposely caused. The need to strengthen existing and develop new partnerships is clear.

Since late 1992, a number of large, complex outbreaks have occurred in the United States. These include the epidemic of over 400,000 cases of waterborne cryptosporidiosis in Milwaukee, the outbreak of severe, unexplained acute respiratory disease now known as hantavirus pulmonary syndrome in the Spring of 1993, the nationwide foodborne salmonellosis outbreak caused by contaminated ice cream that accounted for an estimated 250,000 cases in the fall of 1994, and the increasing problems posed by antimicrobial-resistant organisms in community and health-care settings. Epidemics of plague in India, Ebola hemorrhagic fever in Central Africa, avian (H5N1) influenza in Hong Kong, Hendra virus infection in Australia, and Nipah virus infection recently in Malaysia and Singapore required an international response. During the hantavirus, plague, and Ebola investigations, concerns regarding the possibility of bioterrorism were raised early in the investigations, though these concerns were not supported by subsequent findings.

Investigating these outbreaks in collaboration with local, national, and international partners has provided a number of important lessons, which are reinforced by the threat of bioterrorism. We must avoid complacency and stress preparedness through careful planning and testing of emergency response plans. There is a critical need to strengthen surveillance systems and epidemiologic and laboratory capacity in clinical and public health settings. The outbreaks have illustrated disruptions of travel and commerce and potential threats to national security. The complications of naturally occurring, complex epidemics underline the global implications of local problems. These lessons are directly relevant to the threat of bioterrorism. The challenges of recognizing disease resulting from the clandestine release of an infectious agent are considerable, given the potential for geographic dispersion of the agent (through travel) during the incubation period. The public health approach to bioterrorism must begin with the development of local and state plans formulated collaboratively by the public health, emergency response, and law enforcement communities, which must work together closely in this phase if an epidemic is to be detected in a timely manner, which is critical to its appropriate management. Local health departments and health-care workers will be on the front lines in detection and response. Infection control practitioners, emergency department personnel, microbiologists, first responders, emergency management personnel, and local, state, and federal law enforcement personnel will play vital roles and must engage with each other during the planning stage. Close collaboration between the clinical and public health communities will also be critical.

From a public health perspective, timely surveillance, clinician awareness of syndromes potentially resulting from bioterrorism, epidemiologic investigation capacity, laboratory diagnostic capacity in both clinical and public health laboratory settings, and the ability to rapidly communicate critical information at the local level to those who have a need to know and to manage public communication through the media will be vital. In addition, ensuring the timely availability of an adequate supply of antimicrobial drugs, antitoxins, and vaccines is a formidable challenge. Deployment and administration of stockpiled components to those affected or at greatest risk are also critical.

Address for correspondence: James M. Hughes, National Center for Infectious Diseases, Centers for Disease Control and Prevention, 1600 Clifton Rd NE, Mail Stop C12, Atlanta, GA 30333, USA; fax: 404-639-3039; e-mail: jmh2@cdc.gov.

Recognition of the need for local, regional, and national preparedness for bioterrorism provides an opportunity to strengthen the public health system and its linkages with current and new partners. As President Bill Clinton said in his address at the National Academy of Sciences in January 1998, "These cutting edge efforts will address not only the threat of weapons of mass destruction, but also the equally serious danger of emerging infectious diseases. So we will benefit even if we are successful in avoiding these attacks"(1).

### References

1. Clinton WJ. Remarks by the President on keeping America secure for the 21st Century. National Academy of Sciences, Washington, D.C., January 22, 1999.

